# Subclinical finding in the perception of tactile sensation involvement after SARS-CoV2 infection: comparison with healthy controls using Semmes–Weinstein monofilament testing

**DOI:** 10.3389/fneur.2023.1275063

**Published:** 2023-11-24

**Authors:** Yan Tereshko, Chiara Viotto, Christian Lettieri, Francesca Larese Filon, Enrico Belgrado, Giovanni Merlino, Massimo Bovenzi, Mariarosaria Valente, Gian Luigi Gigli, Simone Dal Bello, Corrado Negro, Federico Ronchese

**Affiliations:** ^1^Clinical Neurology Unit, Udine University Hospital, Udine, Italy; ^2^Department of Medicine (DAME), University of Udine, Udine, Italy; ^3^Department of Medical Sciences, Clinical Unit of Occupational Medicine, University of Trieste, Trieste, Italy; ^4^Neurology Unit, Udine University Hospital, Udine, Italy

**Keywords:** COVID-19, SARS-CoV2, Semmes–Weinstein, sensibility, PACS

## Abstract

**Background:**

Post-acute COVID-19 syndrome patients complain of sensory alterations, mainly positive symptoms such as paresthesia or neuropathic pain but also decreased tactile sensation. Using the Semmes–Weinstein monofilament test (SWMT), our study aims to confront recently infected SARS-CoV2 subjects with a control group.

**Methods:**

This is a cross-sectional, single-centric study. We performed the SWMT (North Coast Medical Inc.) on 30 patients with previous SARS-CoV2 infection (COVID group) and 46 controls (control group). These patients did not present comorbidities or sensory impairment and did not take any medications. The control group tested negative for SARS-CoV2 infection since the COVID-19 pandemic; the COVID group was examined for this study after the resolution of the infection. We tested the threshold of tactile sensation of the tips of the thumb, index, and little finger of each hand, one hand at a time; the dorsum and the hypothenar regions were also tested.

**Results:**

Both groups presented the perception of tactile sensation within the reference value. Despite this result, subclinical changes suggestive of the involvement in peripheral sensory nerve function have been identified in the tested sites in the COVID group compared to the control group. The overall mean target force (grams) was higher in the COVID group than in the control group: 27 (7) vs. 19 (10) mg, *p* < 0.001.

**Conclusion:**

Controls and the COVID group infection had normal tactile sensation thresholds. However, the COVID group presented a higher threshold than the control group, suggesting a possible subclinical perception of tactile sensation involvement of A-beta nerve fibers.

## 1 Introduction

The SARS-CoV2 infection (1) has been correlated to neurological manifestations such as neuropathy, myopathy, Guillain–Barré syndrome, and central nervous system disorders (2–4). Neurological complaints after SARS-CoV2 infection resolution are common in studies, and the term post-acute COVID-19 syndrome (PACS) has been introduced to define this clinical entity. Peripheral nerve system involvement in PACS has been described in the studies (5); the most common symptoms are the persistence of anosmia and ageusia followed by pain, muscle atrophy, paresthesia, hypoesthesia, and numbness (6). In a case series of 100 patients evaluated 4 weeks after Sars-Cov2 infection, 38% presented diffuse paresthesias and 15% had other positive sensory alteration (7). However, little is known about this syndrome and its pathophysiological substrate. Despite the sensory symptoms, most patients had normal sensory nerve conduction studies (8). There is a possibility that subclinical alterations could not be detected with sensory nerve conduction studies. Various non-invasive tests were used to study COVID-19 patients, and the Semmes–Weinstein monofilament (SMWT) test has been used in four adult hospitalized diabetic patients with concomitant severe SARS-CoV2 infection (9). Moreover, the sensory symptoms attributed to the PACS seem unrelated to the gravity of the previous infection (7). Interestingly, a recent case–control study on children and adolescents with previous SARS-CoV2 infection reported increased thermal and vibration thresholds; the mechanical detection threshold with SWMT did not differ from the control group, but there was a significant difference within the SARS-CoV2 group when the patients with previous symptomatic infection and those with asymptomatic infection were compared (10). These studies highlight the involvement of small-diameter sensory fibers (C-fibers and A-delta fibers) due to SARS-CoV2 infection; moreover, the involvement of large sensory fibers was evident only when they compared the patients with previous symptomatic SARS-CoV2 infection to those who had asymptomatic positivity for the virus. This may imply that the subclinical involvement of large sensory fibers may be present even with mild SARS-CoV2 infection; therefore, paresthesias and other positive symptoms may result from this damage. Our research aims to study the perception of tactile sensation, and therefore, the A-beta fibers, with SWMT in healthy adult patients with previous mild SARS-CoV2 infection and to compare the response to healthy controls with no previous infection.

## 2 Methods

### 2.1 Study design and participants

This is a cross-sectional, single-center study. We screened the healthcare professionals of Azienda Sanitaria Universitaria Giuliano Isontina (ASUGI) and proposed the SWMT. We recruited 30 patients with previous SARS-Cov2 infection and tested them with SWMT 3 months after the disease (COVID group); similarly, we recruited 46 controls without a previous SARS-CoV2 infection and tested them with SWMT as well (control group). The controls presenting any comorbidities or taking any medications were excluded. The patients in the COVID group denied sensory symptoms during or after the infection; their SARS-CoV2 infection was mild. According to the protocol for health personnel, the control group was tested with RT-PCR every 2 or 3 weeks since the epidemic. Occasional alcohol drinkers were allowed, while heavy drinkers were excluded in both groups since alcohol can damage sensory fibers. Moreover, we identified the smoker since smoking can damage the sensory system. The results were always negative for SARS-Cov2 infection. Complete neurological examination was negative in both groups. In total, 46 subjects (31 female and 15 male subjects) formed the control group, while 30 subjects (16 female and 14 male subjects) formed the COVID group. Demographic features of the two groups were analyzed, as shown in Table 1, showing no differences between the two groups.

**Table 1:** Demographic features of both groups.

**Variable**	**COVID group (30 patients)**	**Control group (46 patients)**	***p-*value**
Age	37.967 (13.835)	32.870 (11.596)	0.109
BMI	23.442 (5.271)	22.825 (2.847)	0.112
Female	16 (53.33%)	31 (67.39%)	0.218
Smoking	15 (50.00%)	21 (45.65%)	0.815
Occasional alcohol consumption	10 (33.33%)	9 (19.57%)	0.175

There were no differences in age, BMI, sex, smoking, or alcohol assumption. A chi-square test was used to compare the groups; significance was set with a *p*-value of 0.05. The continuous data are described as mean (SD) and the qualitative data as number (percentage).

### 2.2 Ethical aspects

The study was conducted in accordance with the Declaration of Helsinki and approved by our local Ethics Committee approved this study (CEUR n. 092/2018).

All the patients formally consented to the SWMT and data collection for research purposes.

### 2.3 Semmes–Weinstein monofilament test

**Figure 1:**
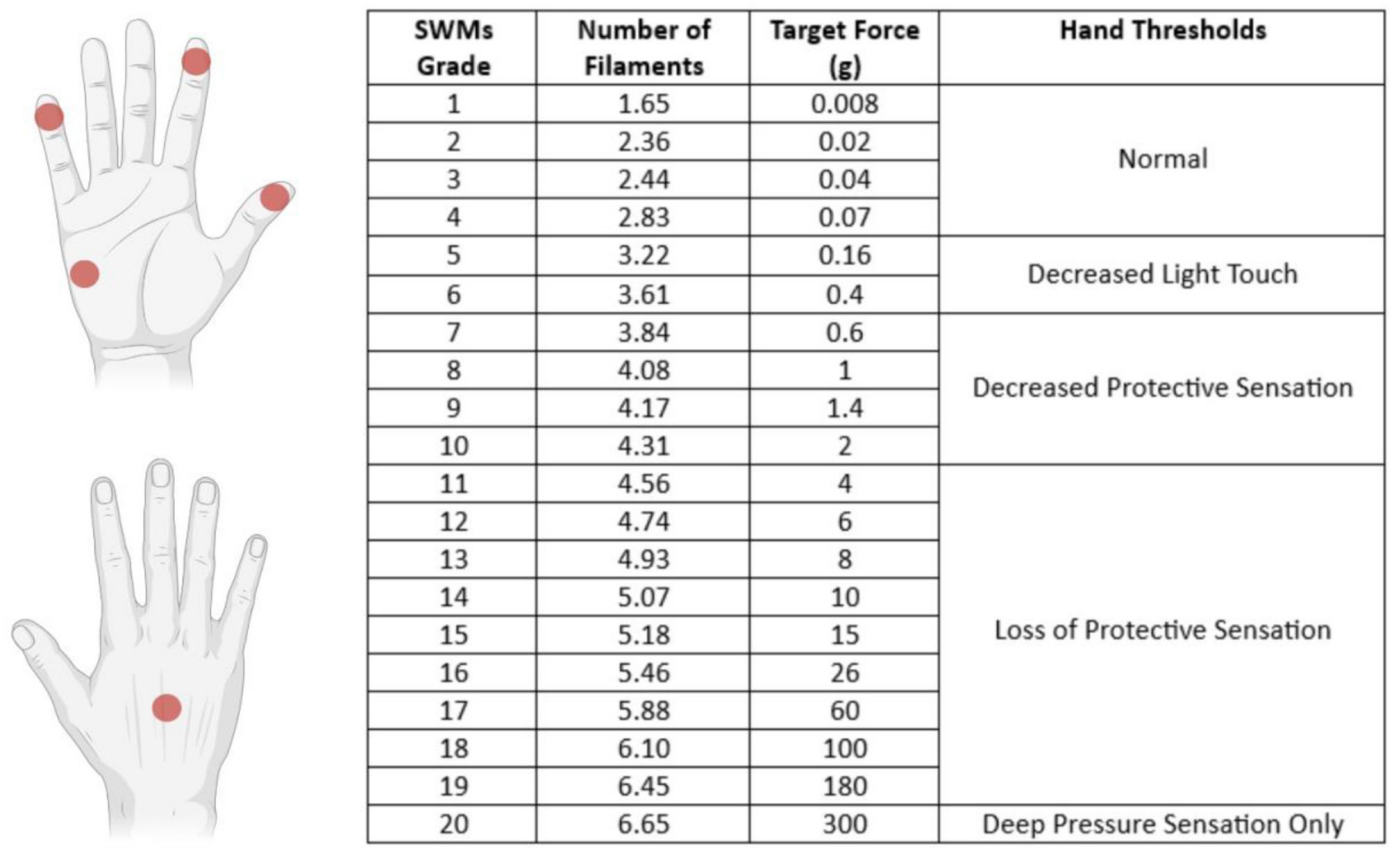
Sites tested with SWMT and each filament's target force (in grams) with the definition of thresholds of perception of tactile sensation. The tip of the I and II fingers are innervated by the median nerve (C6/C7 dermatomes), the V finger is innervated by the ulnar nerve (C8/C7 dermatomes), the hypothenar region is innervated by the palmar branch of the ulnar nerve (C8 dermatome), and the dorsum was tested in the region innervated by the radial nerve (C7 dermatome).

The SWMT was performed by the same operator (CV) in blind, with the subject seated on the opposite side of the testing table. The subjects were tested in a quiet room; each hand was supinated and rested on the surface of the table, and the subject's vision was occluded with a curtain. The subject was instructed to stay with the eyes closed and respond yes when they felt touched. SWMs (North Coast Medical Inc., Morgan Hill) were used to measure the threshold of perception of tactile sensation of the tips of the thumb, index, and little finger of each hand, one hand at a time; the dorsum and the hypothenar regions were also tested. SWMs were defined as grades from 1 (No. 1.65) to 20 (No. 6.65); the lightest filament that could be perceived was defined as the smallest perceivable grade (SPG) (Figure 1). The examiner pressed each filament slowly with a perpendicular angle toward the region's skin tested until it bowed and held it in place for 1.5 s before removing it. The procedure started with the 2.83 filaments, and if it was felt, the examiner applied lighter filaments in a decreasing sequence until one was not felt. If the 2.83 filaments were not felt, the examiner would use thicker filaments in an increasing sequence until one was felt. For filaments from 1.65 to 2.83, this procedure was repeated three times. The examiner then recorded the lighter filament (SPG) that the patient felt.

### 2.4 Endpoint

Our study aimed to research whether subjects with recent SARS-CoV2 infection presented a perception of tactile sensation impairment using the SWMT compared to a control group. We then compared the right and the left hand in the COVID group to assess a possible asymmetric alteration of A-beta fibers.

### 2.5 Data and statistical analysis

A descriptive analysis of the study population's demographics was performed using mean (SD) for continuous variables and numbers and percentages for categorical variables (*n*%). Mean (SD) was used for continuous data. A Shapiro–Wilk test was used to assess the normal distribution of data. For each patient, we calculated the mean target force in both hands and for each hand as follows: we summed the target force obtained with each filament in the different sites, and then we divided the total by the number of the sites tested. Group comparisons were performed as appropriate using a *t*-test or Mann–Whitney's test as appropriate. We employed the chi-square test to compare qualitative variables. All analyses used Stata/SE (version 15.1, StataCorp) for Mac OS. All two-tailed statistical significance levels were set at a *p*-value of < 0.05.

## 3 Results

In both groups, the perception of tactile sensation threshold in every subject was lower or equal to 2.83 filaments in every district examined; these data are consistent with a normal sensory response. However, we found that the COVID group rarely perceived the 1.65 filaments in each district studied (only two subjects perceived the 1.65 filaments on the dorsum of both hands), and their SPG was higher than the control group (Table 2). Specifically, their SPG in the dorsum of the hands was mostly determined by the 2.36 filaments, while 2.44 or 2.36 filaments mostly determined the threshold in the other examination sites.

**Table 2:** Results from the SWMT in controls and COVID groups.

	**1.65 filaments**		***p***	**2.36 filaments**		***p***	**2.44 filaments**		***p***	**2.83 filaments**		***p***
	**COVID**	**Control**		**COVID**	**Control**		**COVID**	**Control**		**COVID**	**Control**	
I Finger left	0 (0%)	17 (36.95%)	**<0.001**	16 (53.33%)	23 (50.00%)	0.818	14 (46.67%)	3 (6.52%)	**<0.001**	0 (0%)	3 (6.52%)	0.274
I Finger right	0 (0%)	8 (17.39%)	**0.019**	19 (63.33%)	29 (63.04%)	0.980	11 (36.67%)	5 (10.87%)	**0.010**	0 (0%)	4 (8.69%)	0.149
II Finger left	0 (0%)	14 (30.43%)	**<0.001**	17 (56.67%)	27 (58.70%)	0.861	13 (43.33%)	3 (6.52%)	**<0.001**	0 (0%)	2 (4.35%)	0.516
II Finger right	0 (0%)	12 (26.08%)	**0.002**	15 (50.00%)	28 (60.87%)	0.350	14 (46.67%)	3 (6.52%)	**<0.001**	1 (3.33%)	3 (6.52%)	0.543
V Finger left	0 (0%)	16 (44.78%)	**<0.001**	17 (56.67%)	27 (68.70%)	0.861	13 (43.33%)	1 (2.17%)	**<0.001**	0 (0%)	2 (4.35%)	0.516
V Finger right	0 (0%)	12 (26.09%)	**0.002**	17 (56.67%)	29 (63.04%)	0.578	11 (36.67%)	2 (4.35%)	**<0.001**	2 (6.67%)	3 (6.52%)	0.980
Dorsum left	2 (6.67%)	33 (71.74%)	**<0.001**	27 (90.00%)	7 (15.22%)	**<0.001**	1 (3.33%)	4 (8.70%)	0.357	0 (0%)	2 (4.35%)	0.516
Dorsum right	2 (6.67%)	32 (69.57%)	**<0.001**	27 (90.00%)	10 (21.74%)	**<0.001**	1 (3.33%)	2 (4.35%)	0.824	0 (0%)	2 (4.35%)	0.516
Hypotenar left	0 (0%)	16 (43.78%)	**<0.001**	19 (63.33%)	26 (56.52%)	0.555	11 (36.67%)	2 (4.35%)	**<0.001**	0 (0%)	2 (4.35%)	0.516
Hipothenar right	0 (0%)	10 (21.74%)	**0.005**	19 (63.33%)	32 (69.57%)	0.765	11 (36.67%)	1 (2.17%)	**<0.001**	0 (0%)	3 (6.52%)	0.274

The SPG was in the normality range in every subject. There was clear evidence of a higher SPG in the COVID group: the minimum threshold was obtained with 2.36 or 2.44 filaments, and only two patients had a threshold of 1.65, which was present only on the dorsum of the hand. The comparison between groups was performed with Pearson's chi-square or Fisher's exact tests as appropriate for each region and side tested; the significance was set with a *p*-value of 0.05. Bold values represent statistical significance.

**Figure 2:**
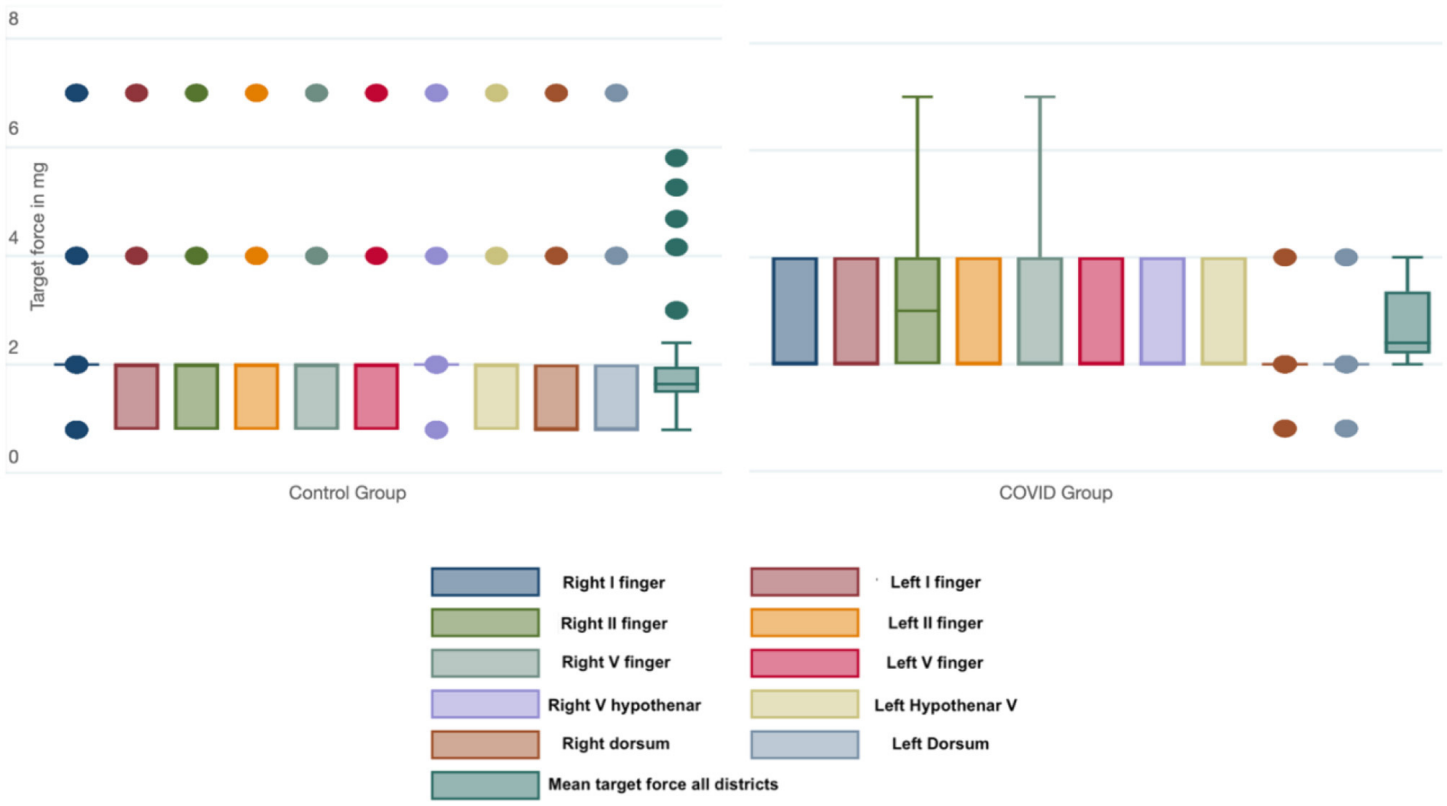
Box-and-whiskers plot of the force (in mg) needed to evoke a sensory response in each district and considering all the districts tested in the Control and COVID groups. The dots represent the outliers.

The mean target force, in milligrams, needed to evoke the sensory response was also calculated for every district. The COVID group presented a significantly higher threshold in every site when compared with the control group (Table 3). We also calculated the mean force, in milligrams, needed to evoke a sensory response in the hands in both groups; this threshold was significantly lower in the control group than in the COVID group, as shown in Figure 2 [19 (11) vs. 27 (7) milligrams; *p* < 0.001].

**Table 3:** The median, in milligrams, of the force needed to evoke light touch sensory response in every district examined for both groups.

	**COVID group**	**Control group**	***p***
	**Target force in mg (mean; SD)**	**Target force in mg (mean; SD)**	
I Finger left	29 (10)	20 (16)	<0.001
I Finger right	27 (10)	24 (16)	0.034
II Finger left	29 (10)	20 (14)	<0.001
II Finger right	27 (10)	21 (15)	<0.001
V Finger left	29 (10)	18 (13)	<0.001
V Finger right	31 (14)	21 (15)	<0.001
Dorsum left	20 (5)	15 (15)	<0.001
Dorsum right	20 (5)	15 (14)	<0.001
Hypothenar left	27 (10)	19 (13)	<0.001
Hipothenar right	27 (10)	21 (14)	<0.001
Mean target force in all districts	27 (7)	19 (11)	<0.001

Comparison between groups was performed with the Mann–Whitney's test (non-normal data distribution); significance was set with a *p*-value of 0.05.

The comparison between the left [27 (7) mg] and right hand [27 (8) mg] in the COVID group did not show any statistical differences (*p* = 0.648).

## 4 Discussion

Sensory symptoms suggestive of peripheral nervous system involvement are commonly reported by COVID-19 patients (11–13). PACS patients frequently complain of sensory alterations, mainly positive symptoms such as paresthesia or neuropathic pain, but also decreased sensation (7, 14). Sensory nerve conduction studies did not report differences between healthy controls and COVID-19 patients although evident impairment was found in severe cases (4, 15–17). Moreover, a recent study reported that patients with previous SARS-CoV2 infection presented subclinical sensory trigeminal impairment for almost 2 years after new-onset olfactory disorder related to COVID-19 (18). The pathophysiological mechanisms of peripheral nerve involvement are still unclear. Sensory fibers could be a potential target for SARS-CoV2, as suggested by other studies in which coronaviruses and other neurotrophic respiratory viruses infested peripheral nervous fibers (19, 20). Some authors hypothesized that olfactory, gustative, and trigeminal terminals could be an entry route for SARS-CoV2 through the retrograde axonal transport mechanism (6, 21, 22). Peripheral nerve involvement could also be through blood–nerve barrier crossing, by the infection of the endothelium, or through infected immune system cells circulating in the bloodstream (6, 21). SARS-CoV2 infection causes the release of inflammatory cytokines such as IL-1, IL6, interferons, and TNF (21); histopathological post-mortem studies of patients that died from SARS-CoV2 infection showed perivascular and/or endoneural inflammatory cells, and overexpression of myxovirus resistance protein A suggesting nerve damage due to inflammatory cytokines (23).

Moreover, SARS-CoV2 can bind ACE2 receptors in the dorsal root ganglion (DRG) of humans; the virus could enter the sensory neurons of the DRG through this receptor and damage their axons (24, 25). SARS-CoV2 proteins have been detected in cranial nerves, brainstem, olfactory bulb, and frontal lobe cells (26, 27) although the clinical significance of these findings is still unclear. Other authors suggested molecular mimicry between SARS-CoV2 proteins and self-antigens, causing the production of autoantibodies that cross-react with neuronal antigens and determining nerve damage. However, there is still no evidence to support molecular mimicry as a possible mechanism of peripheral nerve damage (28, 29). Our subjects had no comorbidities and did not complain of any sensory impairment before the SARS-CoV2 infection and after that. Our study showed that both groups presented normal perception of tactile sensation in both hands according to the SWMT. However, we found a significant difference in the SPG in every site tested: the COVID group presented a higher threshold than the control group. Moreover, there were no differences between the right and left hand in the COVID group. These data suggest a diffuse decrease in the perception of tactile sensation in the COVID group despite the absence of sensory symptoms.

SWMT evaluation was performed in several diseases in which a symmetric alteration of the A-beta fibers has been found, including diabetic neuropathy (30), leprosy (31), Charcot–Marie–Tooth disease (32), but also in focal dystonia (33) and systemic sclerosis (34). In these cases, the threshold in the perception of tactile sensation was not within the normal range as in our study.

It is possible to hypothesize that SARS-CoV2 infection has caused subclinical damage to sensory fibers. The pattern of alteration of the perception of tactile sensation was bilateral and symmetric; therefore, it is suggestive of polyneuropathy. Moreover, it is unknown if these findings are temporary or permanent.

The relevance of our findings has several limitations. First of all, the study is of cross-sectional design; our sample was not studied with SWMT before SARS-CoV2 infection, so we do not know if the subclinical differences undeniably correlate to the infection. The assessment of small-caliber fibers (C-fibers and A-delta fibers) with QST and LEP was not performed. Moreover, we did not perform nerve conduction studies or neuropathological correlations in our sample. Unfortunately, we did not perform this evaluation on the lower limbs. Finally, the sample size is relatively small, and further studies with larger samples and prospective designs are needed to confirm our results.

## 5 Conclusion

In our study, subjects with previous COVID-19 and control subjects had normal perception of tactile sensation thresholds with SWMT. However, the latter group presented a higher threshold than the control group, suggesting a possible subclinical sensory involvement of peripheral nerve fibers.

## Data availability statement

The raw data supporting the conclusions of this article will be made available by the authors, without undue reservation.

## Ethics statement

The studies involving humans were approved by Comitato Etico Unico Regionale (CEUR) del Friuli Venezia Giulia. The studies were conducted in accordance with the local legislation and institutional requirements. The participants provided their written informed consent to participate in this study. Written informed consent was obtained from the individual(s) for the publication of any potentially identifiable images or data included in this article.

## Author contributions

YT: Writing—original draft, Conceptualization, Data curation, Formal analysis, Investigation, Visualization, Writing— review & editing, Methodology, Software, Validation. CV: Conceptualization, Data curation, Formal analysis, Investigation, Methodology, Writing—original draft. CL: Supervision, Validation, Writing—review & editing. FF: Conceptualization, Funding acquisition, Investigation, Methodology, Project administration, Resources, Supervision, Validation, Writing—review & editing. EB: Formal analysis, Validation, Writing—review & editing. GM: Formal analysis, Writing—review & editing. MB: Data curation, Formal analysis, Supervision, Methodology, Writing—review & editing. MV: Funding acquisition, Resources, Supervision, Validation, Investigation, Writing—review & editing. GG: Supervision, Validation, Writing—review & editing. SD: Writing—review & editing. CN: Investigation, Methodology, Software, Writing—review & editing. FR: Conceptualization, Funding acquisition, Investigation, Methodology, Project administration, Resources, Supervision, Validation, Writing—review & editing.
